# Amphibians and reptiles of the state of Chihuahua, Mexico, with comparisons with adjoining states

**DOI:** 10.3897/zookeys.658.10665

**Published:** 2017-02-28

**Authors:** Julio A. Lemos-Espinal, Geoffrey R. Smith, Guillermo A. Woolrich-Piña, Alexander Cruz

**Affiliations:** 1 Laboratorio de Ecología-UBIPRO, FES Iztacala UNAM. Avenida los Barrios 1, Los Reyes Iztacala, Tlalnepantla, edo. de México, Mexico – 54090; 2 Department of Biology, Denison University, Granville, OH, USA 43023; 3 Instituto Tecnológico Superior de Zacapoaxtla. Carretera Acuaco-Zacapoaxtla Km. 8, Col. Totoltepec C. P. 73680, Zacapoaxtla, Puebla, Mexico; 4 Department of Ecology and Evolutionary Biology (EBIO), University of Colorado - Boulder, Campus Box 334 UCB, Boulder, CO USA 80309-0334

**Keywords:** Checklist, Chihuahuan Desert, conservation status, herpetofauna, Sierra Madre Occidental

## Abstract

Chihuahua is Mexico’s largest state, and its physiographic complexity affects the distribution of its herpetofauna. We list amphibians and reptiles for the state of Chihuahua, with their conservation status. We also compare this list to those of six adjoining states in the United States and Mexico (New Mexico, Texas, Coahuila, Durango, Sinaloa, and Sonora). A total of 175 species of amphibians and reptiles is found in Chihuahua. Thirty-eight are amphibians, and 137 reptiles. Chihuahuan amphibians and reptiles represent just over 37% of such species from Chihuahua and neighboring states. Chihuahua shares the highest proportion of its herpetofauna with Sonora and Durango. Most of the herpetofauna of Chihuahua falls in IUCNs least concern category and is not listed by SEMARNAT. However, turtles in Chihuahua are a group of particular conservation concern.

## Introduction

**Figure 1.**
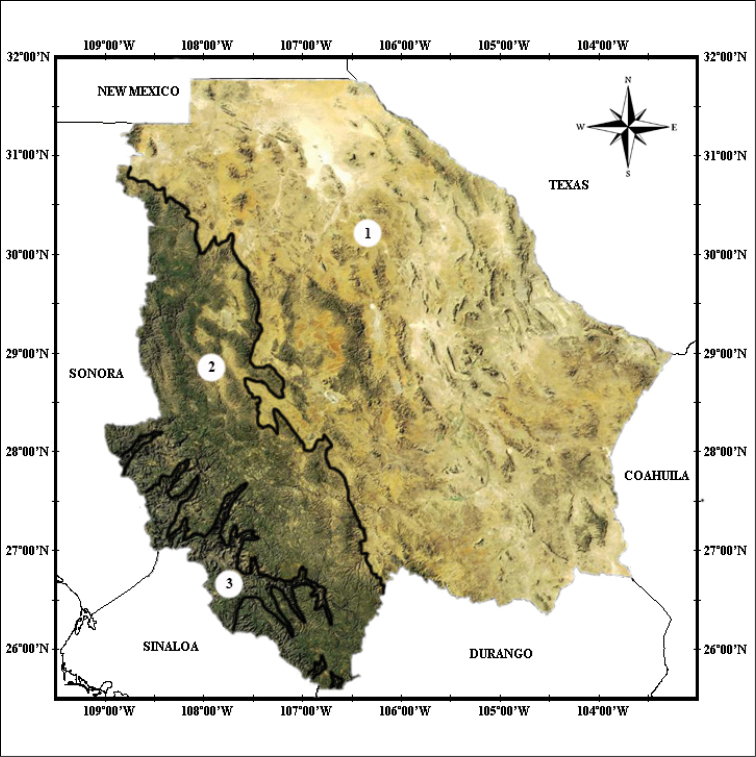
Topographical map of the state of Chihuahua, Mexico: **1** Chihuahuan Desert **2** Sierra Madre Occidental, and **3** Cooper Canyon (INEGI 2001).

Chihuahua is the largest state in Mexico. Its 245,612 km^2^ (lying between 25°38'N to the south, 31°47'N to the north, and between103°18'W to the east, and 109°7'W to the west) represent 12.6% of the total territory of the nation. Chihuahua is physiographically complex (Fig. 1), and this complexity affects the distribution of the herpetofauna.

The western part of the state is primarily occupied by the Sierra Madre Occidental, which passes the Continental Divide, separating the Pacific and Atlantic drainages. In Chihuahua, the Sierra Madre varies in width from ~130–160 km in the south (west of Hidalgo de Parral) to ~65–80 km in the north (west of Casas Grandes) (Tanner 1985, Lemos-Espinal and H. Smith 2007). The topography of the Sierra Madre Occidental of Chihuahua is very heterogeneous. The highest altitude is on Cerro Mohinora, at 3,300 m, and the extreme southwestern Pacific slopes of this Sierra are characterized by deep canyons that drop down to ~250 m in the Barranca del Septentrión/Cañón de Chínipas, making diverse habitats for plants and animals. Copper Canyon is a 64,750 km^2^ system of six interconnected canyons located in Southwestern Chihuahua. Four of these six canyons are deeper than the Grand Canyon, some by over 305 m. The deepest canyon is Urique Canyon, 1,870 m in depth; Batopilas Canyon is 1,830 m deep; Sinforosa Canyon is 1,800 m deep; and Copper Canyon is 1,759 m deep (Martin et al.1998, INEGI 2004, Wyndham 2004, Lemos-Espinal and H. Smith 2007, 2015a, Lemos-Espinal et al. 2013, http://www.earlham.edu/~garcier/Geology/coppercanyon.htm).

Over half of the state of Chihuahua, east of the Sierra Madre, is covered by high plains at ~1,200–1,700 m. From these plains arise a large number of small to medium-sized, isolated sierras, some of which reach altitudes of over 2,000 m. Some are high enough to support coniferous forests, constituting continental “islands” surrounded by a “sea” of semiarid plains, where differentiation among populations is enhanced by isolation.

In extreme northeastern Chihuahua, deep canyons, similar to those on the Pacific side of the Sierra Madre Occidental, cut into the edge of the high plains, and support their own distinct herpetofaunal assemblages. Among them is the great Cañón de Santa Elena, in the Zona de Protección de Flora y Fauna Silvestre Cañón de Santa Elena, an extension of the Big Bend National Park of the United States.

In recent years, there has been a considerable increase in the study of Mexican regional and state herpetofaunas such as Sinaloa (Hardy and McDiarmid 1969), Peninsula of Baja California (Grismer 2002), Peninsula of Yucatan (Lee 1996), the Valley of Mexico (Ramírez-Bautista et al. 2009), Aguascalientes (McCranie and Wilson 2001), Chihuahua and Coahuila (Lemos-Espinal and H. Smith 2007a, b, 2015a, b; Lemos-Espinal and G. Smith 2016), Querétaro (Dixon and Lemos-Espinal 2010), San Luis Potosí (Lemos-Espinal and Dixon 2013), Michoacán (Alvarado-Díaz et al. 2015), Chiapas (Johnson et al. 2015b), Oaxaca (Mata-Silva et al. 2015), Nayarit (Woolrich-Piña et al. 2016), Nuevo León (Lemos-Espinal et al. 2016), Sonora (Enderson et al. 2009, Lemos-Espinal and Rorabaugh 2015, Lemos-Espinal et al. 2015, Rorabaugh 2008, Rorabaugh and Lemos-Espinal 2016), and Tamaulipas (Farr 2015, Terán-Juárez et al. 2016).

Among these states Chihuahua has received a great deal of attention in the study of its herpetofauna. Lemos-Espinal and H. Smith (2015a) reviewed herpetological studies previously done in this state, reporting a total of 158 publications related to amphibian and reptile species since the description of *Axolotes maculata* (= *Ambystoma rosaceum*) by Owen (1844) through the description of *Incilius mccoyi* by Santos-Barrera and Flores-Villela (2011), adding the recent publications by Anderson and Greenbaum (2012), Villa et al. (2012), Uriarte-Garzón and García-Vázquez (2014), Lemos-Espinal and H. Smith (2015a), and Lemos-Espinal et al. (2015). The number of publications has increased to 163. The chronological distribution of these publications is the following: prior to 1850 (1); 1851–1875 (4); 1876–1900 (5); 1901–1925 (1); 1926–1950 (14); 1951–1975 (18); 1976–2000 (37); 2001–2015 (83), suggesting a surge in interest and knowledge about the herpetofauna of Chihuahua.

Although there has been a considerable interest in the herpetofauna of Chihuahua, as stated above, none of these 163 publications has focused on the conservation statuses of the documented species for this state. Here, we report the list of amphibians and reptiles that have been recorded so far for the state of Chihuahua. While checklists for Chihuahua are available (e.g., Lemos-Espinal and H. Smith 2007, 2015a), we expand on these earlier efforts by also collecting and summarizing the conservation statuses for each documented species. We also compare the list of the six adjoining states in the United States and Mexico for which recent checklists are available (New Mexico, Texas, Coahuila, Durango, Sinaloa, and Sonora). Our goal is to place this checklist into a regional and conservation context not available in previously published checklists.

## Methods

We compiled the list of amphibians and reptiles of the state of Chihuahua from the following sources: (1) our own field work; (2) specimens from the Laboratorio de Ecología – UBIPRO (**LEUBIPRO**) collections; (3) databases from the Comisión Nacional para el Conocimiento y Uso de la Biodiversidad (National Commission for the Understanding and Use of Biodiversity; **CONABIO**), including records from the following 22 collections Colección Herpetológica, Departamento de Zoología, Escuela Nacional de Ciencias Biológicas (**ENCB**); Colección Herpetológica, Museo de Zoología “Alfonso L. Herrera”, Facultad de Ciencias UNAM (**MZFC**); Colección Nacional de Anfibios y Reptiles, Instituto de Biología UNAM (**CNAR**); Amphibians and Reptiles Collection, University of Arizona (**UAZ**); Collection of Herpetology, Amphibians and Reptiles Section, Carnegie Museum of Natural History, Pittsburgh; Collection of Herpetology, Biology Department, Tulane University, New Orleans (**TU**); Collection of Herpetology, Department of Vertebrate Zoology, National Museum of Natural History, Smithsonian Institution (**USNM**); Collection of Herpetology, Herpetology Department, American Museum of Natural History (**AMNH**); Collection of Herpetology, Herpetology Department, California Academy of Sciences (**CAS**); Collection of Herpetology, Museum of Comparative Zoology, Harvard University Cambridge (**MCZ**); Collection of Herpetology, Museum of Vertebrate Zoology, Division of Biological Sciences, University of California Berkeley (**MVZ**); Collection of Herpetology, Museum of Zoology, University of Michigan Ann Arbor (**UMMZ**); Collection of Herpetology, Texas Cooperative Wildlife Collection, Texas A&M University (**TCWC**); Collection of Herpetology, Texas Natural History Collection, University of Texas Austin (**TNHC**); Collection of Herpetology, University of Colorado Museum (**UCM**); Collection of Herpetology, University of Illinois Museum of Natural History (**UIMNH**); Division of Amphibians and Reptiles, Field Museum of Natural History (**FMNH**); Fort Worth Museum of Sciences and History (**FWMSH**); Herpetology Section, Natural History Museum of Los Angeles County (**LACM**); Louisiana State University, Museum of Life Sciences; Merriam Museum, University of Texas Arlington (**UTAMM**); Museum of Natural History, Division of Herpetology, Kansas University (**MNHUK**); and (4) a thorough examination of the available literature on amphibians and reptiles in the state. Species were included in the checklist only if we were able to confirm the record, either by direct observation or through documented museum records or vouchers in the state. In addition, we recorded the conservation status of each species based on three sources: 1) the IUCN Red List, 2) Environmental Viability Scores from Wilson et al. (2013a,b), and 3) listing in SEMARNAT (2010).

Scientific names used in this publication are based on the taxonomic list published in Lemos-Espinal (2015). The amphibian names follows Frost (2016) and the reptile names follows Uetz and Hošek (2016). State lists used to compare the species composition between Chihuahua and the adjoining states were: Dixon (2015) for Texas; Enderson et al. (2009) for Sinaloa; Lemos-Espinal and G. Smith (2016) for Coahuila; Painter and Stuart (2015) for New Mexico; Rorabaugh and Lemos-Espinal (2016) for Sonora; and Valdez-Lares et al. (2013) for Durango. We updated these lists for Coahuila (adding *Crotalus ornatus*, Nevárez De los Reyes et al. [2016]); Sonora and Sinaloa (adding *Gopherus evgoodei*, Edwards et al. [2016]); Texas (adding *Crotalus ornatus*, Anderson and Greenbaum [2012]); Durango (we regarded the population of *Barisia imbricata* [Wiegmann] as *Barisia ciliaris* [Smith]; *Sceloporus edbelli* Smith et al. as part of *Sceloporus consobrinus* Baird & Girard; *Sceloporus lineolateralis* Smith as part of *Sceloporus jarrovii* Cope; and *Aspidoscelis scalaris* [Baird & Girard] as part of *Aspidoscelis gularis* [Baird & Girard]). We also determined the number of overlapping species between each of these states and Chihuahua.

## Results and discussion

A total of 175 (173 native, two introduced) species of amphibians and reptiles is found in Chihuahua. Thirty-eight of these species are amphibians (four salamanders, 34 anurans [one introduced]), and 137 are reptiles (13 turtles, 51 lizards [one introduced], and 73 snakes) (Tables 1, 2). These represent 32 families: nine amphibians (two salamanders; seven anurans), and 23 reptiles (five of turtles, 11 of lizards and seven of snakes), and 81 genera: 16 amphibians (two salamanders, 14 anurans), and 65 reptiles (seven of turtles, 20 of lizards and 38 of snakes). The introduced species are the American Bullfrog (*Lithobates catesbeianus*) and the Mediterranean House Gecko (*Hemidactylus turcicus*).

**Table 1.** Checklist of amphibians and reptiles of Chihuahua providing the habitat type (CD = Chihuahuan Desert, SMO = Temperate Forests of the Sierra Madre Occidental, SBT = Subtropics – Canyons of the Sierra Madre Occidental; GEN = Generalist – occupies more than one habitat type), IUCN Status (DD = Data Deficient; LC = Least Concern, V = Vulnerable, NT = Neat Threatened; E = Endangered; CE = Critically Endangered) according to the IUCN Red List (The IUCN Red List of Threatened Species, Version 2016.1; www.iucnredlist.org; accessed 30 June 2016), Environmental Vulnerability Score (EVS; the higher the score the greater the vulnerability; NE = not evaluated) from Wilson et al. (2013a,b) and Johnson et al. (2015a), and conservation status in Mexico according to SEMARNAT (2010) (P = in danger of extinction, A = threatened; Pr = subject to special protection, NL – not listed). Source denotes whether the species was observed in the field by the authors (A), documented in the CONABIO data base and/or museum collections (C/M), or found in the literature (citation of source).

	Habitat type	IUCN	EVS	SEMARNAT	Source
**CLASS AMPHIBIA**					
**ORDER CAUDATA**					
**Ambystomatidae**					
*Ambystoma mavortium* Baird	CD	LC	10	NL	A
*Ambystoma rosaceum* Taylor	SMO	LC	14	Pr	A
*Ambystoma silvense* Webb	SMO	DD	14	NL	C/M
**Plethodontidae**					
*Isthmura sierraoccidentalis* (Gray)	SMO	V	12	A ^1^	C/M
**ORDER ANURA**					
**Bufonidae**					
*Anaxyrus cognatus* (Say)	CD	LC	8	NL	A
*Anaxyrus debilis* (Girard)	CD	LC	7	Pr	A
*Anaxyrus mexicanus* (Brocchi)	SMO	NT	13	NL	A
*Anaxyrus punctatus* (Baird & Girard)	GEN	LC	5	NL	A
*Anaxyrus speciosus* (Girard)	CD	LC	12	NL	A
*Anaxyrus woodhousii* (Girard)	GEN	LC	10	NL	A
*Incilius alvarius* (Girard)	CD	LC	11	NL	Santos-Barrera et al. (2006)
*Incilius mazatlanensis* (Taylor)	SBT	LC	12	NL	A
*Incilius mccoyi* Santos-Barrera & Flores-Villela	SMO	NL	14	NL	A
*Rhinella horribilis* (Linnaeus)	SBT	LC	3	NL	A
**Craugastoridae**					
*Craugastor augusti* (Dugès)	SBT	LC	8	NL	C/M
*Craugastor tarahumaraensis* (Taylor)	SMO	V	17	Pr	A
**Eleutherodactylidae**					
*Eleutherodactylus interorbitalis* (Langebartel & Shannon)	SBT	DD	15	Pr	A
*Eleutherodactylus marnockii* (Cope)	CD	LC	NE	NL	A
**Hylidae**					
*Hyla arenicolor* Cope	SMO	LC	7	NL	A
*Hyla wrightorum* Taylor, 1939	SMO	LC	9	NL	A
*Agalychnis dacnicolor* (Cope)	SBT	LC	13	NL	A
*Smilisca baudinii* (Duméril & Bibron)	SBT	LC	3	NL	A
*Tlalocohyla smithii* (Boulenger)	SBT	LC	11	NL	A
**Microhylidae**					
*Gastrophryne mazatlanensis* (Taylor)	SBT	NL	8	NL	A
*Gastrophryne olivacea* (Hallowell)	CD	LC	9	Pr	A
*Hypopachus variolosus* (Cope)	SBT	LC	4	NL	A
**Ranidae**					
*Lithobates berlandieri* (Baird)	CD	LC	7	Pr	A
*Lithobates catesbeianus* (Shaw) – **Introduced**	SMO	LC	10	NL	A
*Lithobates chiricahuensis* (Platz & Mecham)	SMO	V	11	A	A
*Lithobates forreri* (Boulenger)	SBT	LC	3	Pr	A
*Lithobates lemosespinali* (Smith & Chiszar) **Endemic**	SMO	DD	14	NL	A
*Lithobates magnaocularis* (Frost & Bagnara)	GEN	LC	12	NL	A
*Lithobates pustulosus* (Boulenger)	SBT	LC	9	Pr	C/M
*Lithobates tarahumarae* (Boulenger)	SMO	V	8	NL	A
*Lithobates yavapaiensis* (Platz & Frost)	SMO	LC	12	Pr	A
**Scaphiopodidae**					
*Scaphiopus couchi* Baird	GEN	LC	3	NL	A
*Spea bombifrons* (Cope)	CD	LC	10	NL	A
*Spea multiplicata* (Cope)	GEN	LC	6	NL	A
**CLASS REPTILIA**					
**ORDER TESTUDINES**					
**Emydidae**					
*Chrysemys picta* (Schneider)	GEN	LC	14	A	A
*Terrapene nelsoni* Stejneger	SMO	DD	18	Pr	A
*Terrapene ornata* (Agassiz)	CD	NT	15	Pr	A
*Trachemys gaigeae* (Hartweg)	CD	V	18	NL	A
**Geoemydidae**					
*Rhinoclemmys pulcherrima* (Gray)	SBT	NL	8	NL	A
**Kinosternidae**					
*Kinosternon durangoense* Iverson	CD	DD	16	NL	A
*Kinosternon flavescens* (Agassiz)	CD	LC	12	NL	A
*Kinosternon hirtipes* (Wagler)	GEN	LC	10	Pr	A
*Kinosternon integrum* LeConte	SBT	LC	11	Pr	A
*Kinosternon sonoriense* Le Conte	GEN	NT	14	P – subsp longifemorale	A
**Testudinidae**					
*Gopherus flavomarginatus* Legler	CD	V	19	P	A
*Gopherus evgoodei* Edwards, Karl, Vaughn, Rosen, Meléndez-Torres, & Murphy	SBT	NL	NE	A ^2^	A
**Trionychidae**					
*Apalone spinifera* (Le Sueur)	CD	LC	15	Pr	A
**ORDER SQUAMATA**					
**SUBORDER LACERTILIA**					
**Anguidae**					
*Barisia ciliaris* (Smith)	SMO	NL	15	NL	A
*Barisia levicollis* Stejneger **Endemic**	SMO	DD	15	Pr	A
*Elgaria kingii* Gray	SMO	LC	10	Pr	A
*Gerrhonotus infernalis* Baird	SMO	LC	13	NL	A
**Crotaphytidae**					
*Crotaphytus collaris* (Say)	GEN	LC	13	A	A
*Gambelia wislizenii* (Baird & Girard)	CD	LC	13	Pr	A
**Dactyloidae**					
*Anolis nebulosus* (Wiegmann)	SBT	LC	13	NL	A
**Eublepharidae**					
*Coleonyx brevis* Stejneger	CD	LC	14	Pr	A
**Gekkonidae (INTRODUCED)**					
*Hemidactylus turcicus* (Linnaeus) **Introduced**		N/A	N/A	N/A	A
**Helodermatidae**					
*Heloderma horridum* Wiegmann	SBT	LC	11	A	A
**Iguanidae**					
*Ctenosaura macrolopha* Smith	SBT	NL	19	Pr ^1^	A
**Phrynosomatidae**					
*Cophosaurus texanus* Troschel	CD	LC	14	A	A
*Holbrookia approximans* Baird	CD	NL	14	NL	A
*Holbrookia elegans* Bocourt	GEN	LC	13	NL	A
*Holbrookia maculata* Girard	GEN	LC	10	NL	A
*Phrynosoma cornutum* (Harlan)	CD	LC	11	NL	A
*Phrynosoma hernandesi* Girard	SMO	LC	13	NL	A
*Phrynosoma modestum* Girard	CD	LC	12	NL	A
*Phrynosoma orbiculare* (Linnaeus)	SMO	LC	12	A	A
*Sceloporus albiventris* Smith	SBT	NL	16	NL	A
*Sceloporus bimaculosus* Phelan & Brattstrom	CD	NL	NE	NL	A
*Sceloporus clarkii* Baird & Girard	GEN	LC	10	NL	A
*Sceloporus consobrinus* Baird & Girard	CD	NL	NE	NL	A
*Sceloporus cowlesi* Lowe & Norris	CD	NL	13	NL	A
*Sceloporus jarrovii* Cope	SMO	LC	11	NL	A
*Sceloporus lemosespinali* Lara-Góngora	SMO	DD	16	NL	A
*Sceloporus merriami* Stejneger	CD	LC	13	NL	A
*Sceloporus nelsoni* Cochran	SBT	LC	13	NL	A
*Sceloporus poinsettii* Baird & Girard	CD	LC	12	NL	A
*Sceloporus slevini* Smith	SMO	LC	11	NL	A
*Sceloporus virgatus* Smith	SMO	LC	15	NL	A
*Uma paraphygas* Williams, Chrapliwy & Smith	CD	NT	17	P	A
*Urosaurus bicarinatus* (Duméril)	SBT	LC	12	NL	A
*Urosaurus ornatus* (Baird & Girard)	GEN	LC	10	NL	A
*Uta stansburiana* Baird & Girard	CD	LC	11	A	A
**Phyllodactylidae**					
*Phyllodactylus tuberculosus* Wiegmann	SBT	LC	8	NL	A
**Scincidae**					
*Plestiodon bilineatus* (Tanner)	SMO	NL	13	NL	A
*Plestiodon callicephalus* (Bocourt)	SMO	LC	12	NL	A
*Plestiodon multilineatus* (Tanner) **Endemic**	SMO	DD	16	Pr	Van Devender and Van Devender (1975)
*Plestiodon multivirgatus* (Hallowell)	CD	LC	14	Pr	A
*Plestiodon obsoletus* (Baird & Girard)	CD	LC	11	NL	A
*Plestiodon parviauriculatus* (Taylor)	SMO	DD	15	Pr	A
*Plestiodon tetragrammus* (Baird)	CD	LC	12	NL	A
**Teiidae**					
*Aspidoscelis costata* (Cope)	SBT	NL	11	Pr	A
*Aspidoscelis exsanguis* (Lowe)	CD	LC	14	NL	A
*Aspidoscelis gularis* (Baird & Girard)	CD	LC	9	NL	A
*Aspidoscelis inornata* (Baird)	CD	LC	14	NL	A
*Aspidoscelis marmorata* (Baird & Girard)	CD	NL	14	NL	A
*Aspidoscelis sonorae* (Lowe & Wright)	SMO	LC	13	NL	A
*Aspidoscelis tesselata* (Say)	CD	LC	14	NL	A
*Aspidoscelis uniparens* (Wright & Lowe)	CD	LC	15	NL	A
**ORDER SQUAMATA**					
**SUBORDER SERPENTES**					
**Boidae**					
*Boa sigma* Daudin	SBT	NL	NE	A ^3^	A
**Colubridae**					
*Arizona elegans* Kennicott	CD	LC	5	NL	A
*Bogertophis subocularis* (Brown)	CD	LC	14	NL	A
*Conopsis nasus* Günther	SMO	LC	11	NL	A
*Drymarchon melanurus* (Duméril, Bibron & Duméril)	SBT	LC	6	NL	A
*Drymobius margaritiferus* (Schlegel)	SBT	NL	6	NL	A
*Gyalopion canum* Cope	CD	LC	9	NL	A
*Gyalopion quadrangulare* (Günther)	SBT	LC	11	Pr	A
*Lampropeltis getula* (Linnaeus)	GEN	LC	NE	A	A
*Lampropeltis knoblochi* Taylor	SMO	NL	10	A ^4^	A
*Lampropeltis polyzona* Cope	SBT	NL	11	NL	A
*Leptophis diplotropis* (Günther)	SBT	LC	14	A	A
*Masticophis bilineatus* Jan	GBN	LC	11	NL	A
*Masticophis flagellum* Shaw	CD	LC	8	A	A
*Masticophis mentovarius* (Duméril, Bibron & Duméril)	SBT	LC	6	A	A
*Masticophis taeniatus* (Hallowell)	GEN	LC	10	NL	A
*Mastigodryas cliftoni* (Hardy)	SBT	NL	14	NL	A
*Opheodrys vernalis* (Harlan)	SMO	LC	NE	NL	Van Devender and Lowe (1977)
*Oxybelis aeneus* (Wagler)	SBT	NL	5	NL	A
*Pantherophis emoryi* (Baird & Girard)	CD	LC	13	NL	A
*Pituophis catenifer* (Blainville)	GEN	LC	9	NL	A
*Pituophis deppei* (Duméril)	SMO	LC	14	A	A
*Rhinocheilus lecontei* Baird & Girard	CD	LC	8	NL	A
*Salvadora bairdii* Jan & Sordelli	SMO	LC	15	Pr	A
*Salvadora deserticola* Schmidt	CD	NL	14	NL	A
*Salvadora grahamiae* Baird & Girard	GEN	LC	10	NL	A
*Senticolis triaspis* (Cope)	SBT	LC	6	NL	A
*Sonora aemula* (Cope)	SBT	NT	16	Pr	A
*Sonora semiannulata* Baird & Girard	GEN	LC	5	NL	A
*Sympholis lippiens* Cope	SBT	NL	14	NL	A
*Tantilla hobartsmithi* Taylor	CD	LC	11	NL	A
*Tantilla nigriceps* Kennicott	CD	LC	11	NL	A
*Tantilla wilcoxi* Stejneger	SMO	LC	10	NL	A
*Tantilla yaquia* Smith	SBT	LC	10	NL	A
*Trimorphodon tau* Cope	SBT	LC	13	NL	A
*Trimorphodon vilkinsonii* Cope	CD	LC	15	A	A
**Dipsidae**					
*Diadophis punctatus* (Linnaeus)	GEN	LC	4	NL	A
*Geophis dugesii* Bocourt	SMO	LC	13	NL	A
*Heterodon kennerlyi* Kennicott	CD	NL	11	Pr	A
*Hypsiglena chlorophaea* Cope	GEN	NL	8	NL	A
*Hypsiglena jani* (Dugès)	CD	NL	6	NL	A
*Imantodes gemmistratus* (Cope)	SBT	NL	6	Pr	A
*Leptodeira splendida* (Günther)	SBT	LC	14	NL	A
*Rhadinaea hesperia* Bailey	SMO	LC	10	Pr - subsp baileyi	A
*Rhadinaea laureata* (Günther)	SMO	LC	12	NL	Villa et al. (2012)
*Tropidodipsas repleta* Smith, Lemos-Espinal, Hartman & Chiszar	SBT	DD	17	NL	A
**Elapidae**					
*Micruroides euryxanthus* (Kennicott)	SON	NL	15	A	A
*Micrurus distans* (Kennicott)	SBT	LC	14	Pr	A
**Leptotyphlopidae**					
*Rena dissecta* (Cope)	CD	LC	11	NL	C/M
*Rena humilis* Baird & Girard	CD	LC	8	NL	A
*Rena segrega* (Klauber)	CD	NL	NE	NL	C/M
**Natricidae**					
*Nerodia erythrogaster* (Forster)	CD	LC	11	A	Uriarte-Garzón and García-Vázquez (2014)
*Storeria storerioides* (Cope)	SMO	LC	11	NL	A
*Thamnophis cyrtopsis* (Kennicott)	GEN	LC	7	A	A
*Thamnophis elegans* (Baird & Girard)	SMO	LC	14	A	A
*Thamnophis eques* (Reuss)	GEN	LC	8	A	A
*Thamnophis errans* Smith	SMO	LC	16	NL	A
*Thamnophis marcianus* (Baird & Girard)	GEN	LC	10	A	A
*Thamnophis melanogaster* (Peters)	SMO	E	15	A	A
*Thamnophis sirtalis* (Linnaeus)	SMO	LC	14	Pr	A
*Thamnophis unilabialis* Tanner	SMO	NL	NE	NL	A
*Thamnophis validus* (Kennicott)	SBT	NL	12	NL	A
**Viperidae**					
*Agkistrodon bilineatus* (Günther)	SBT	NT	11	Pr	A
*Agkistrodon contortrix* (Linnaeus)	CD	LC	14	NL	C/M
*Crotalus atrox* Baird & Girard	CD	LC	9	Pr	A
*Crotalus basiliscus* (Cope)	SBT	LC	16	Pr	A
*Crotalus lepidus* (Kennicott)	SMO	LC	12	Pr	A
*Crotalus molossus* Baird & Girard	GEN	LC	8	Pr	A
*Crotalus ornatus* Hallowell	CD	NL	13	NL	Anderson and Greenbaum (2012)
*Crotalus pricei* Van Denburgh	SMO	LC	14	Pr	A
*Crotalus scutulatus* (Kennicott)	CD	LC	11	Pr	A
*Crotalus viridis* (Rafinesque)	CD	LC	12	Pr	A
*Crotalus willardi* Meek	SMO	LC	13	Pr	A

A^1^ = Protected under the name *Pseudoeurycea bellii*; A^2^ = Protected under the name *Gopherus agassizii*; A^3^ = Protected under the name *Boa constrictor*; A^4^ = Protected under the name *Lampropeltis pyromelana*; Pr^1^ = Protected under the name *Ctenosaura hemilopha*.

**Table 2.** Summary of species present in Chihuahua by family, order or suborder, and class. Status summary indicates the number of species found in each IUCN conservation status in the Order DD, LC, V, NT, E, CE (see Table 1 for abbreviations; in some cases species have not been assigned a status by the IUCN and therefore these may not add up to the total number of species in a taxon). Mean EVS is the mean Environmental Vulnerability Score, scores ≥ 14 are considered high vulnerability (Wilson et al. 2013a,b) and conservation status in Mexico according to SEMARNAT (2010) in the Order NL, Pr, P, A (see Table 1 for abbreviations).

Class	Order/Suborder	Family	Genera	Species	Status Summary	Mean EVS	SEMARNAT
Amphibia	Caudata		**2**	**4**	**1,2,1,0,0,0**	**12.5**	**2,1,1,0**
		Ambystomatidae	1	3	1,2,0,0,0,0	12.7	2,1,0,0
		Plethodontidae	1	1	0,0,1,0,0,0	12	0,0,1,0
	Anura		**14**	**34**	**2,26,3,1,0,0**	**9.8**	**25,8,1,0**
		Bufonidae	3	10	0,8,0,1,0,0	9.5	9,1,0,0
		Craugastoridae	1	2	0,1,1,0,0,0	12.5	1,1,0,0
		Eleutherodactylidae	1	2	1,1,0,0,0,0	15	1,1,0,0
		Hylidae	4	5	0,5,0,0,0,0	8.6	5,0,0,0
		Microhylidae	2	3	0,2,0,0,0,0	7	2,1,0,0
		Ranidae	1	9	1,6,2,0,0,0	9.6	4,4,1,0
		Scaphiopodidae	2	3	0,3,0,0,0,0	6.3	3,0,0,0
	**Subtotal**		**16**	**38**	**3,28,4,1,0,0**	**9.96**	**27,9,2,0**
Reptilia	Testudines		**7**	**13**	**2,5,2,2,0,0**	**14.16**	**5,6,2,0**
		Emydidae	3	4	1,1,1,1,0,0	16.25	1,2,1,0
		Geoemydidae	1	1	0,0,0,0,0,0	8	1,0,0,0
		Kinosternidae	1	5	1,3,0,1,0,0	12.6	3,2,0,0
		Testudinidae	1	2	0,0,1,0,0,0	19	0,1,1,0
		Trionychidae	1	1	0,1,0,0,0,0	15	0,1,0,0
	Squamata						
	Lacertilia		**20**	**51**	**4,34,0,1,0,0**	**12.91**	**35,9,5,1**
		Anguidae	3	4	1,2,0,0,0,0	13.25	2,2,0,0
		Crotaphytidae	2	2	0,2,0,0,0,0	13	0,1,1,0
		Dactyloidae	1	1	0,1,0,0,0,0	13	1,0,0,0
		Eublepharidae	1	1	0,1,0,0,0,0	14	0,1,0,0
		Gekkonidae	1	1			
		Helodermatidae	1	1	0,1,0,0,0,0	11	0,0,1,0
		Iguanidae	1	1	0,0,0,0,0,0	19	0,1,0,0
		Phrynosomatidae	7	24	1,16,0,1,0,0	12.7	20,0,3,1
		Phyllodactylidae	1	1	0,1,0,0,0,0	8	1,0,0,0
		Scincidae	1	7	2,4,0,0,0,0	13.3	4,3,0,0
		Teiidae	1	8	0,6,0,0,0,0	13	7,1,0,0
	Serpentes		**38**	**73**	**1,52,0,2,1,0**	**10.95**	**38,17,16,0**
		Boidae	1	1	0,0,0,0,0,0	?	0,0,1,0
		Colubridae	21	35	0,27,0,1,0,0	10.5	23,3,7,0
		Dipsidae	8	10	1,5,0,0,0,0	10.1	7,3,0,0
		Elapidae	2	2	0,1,0,0,0,0	14.5	0,1,1,0
		Leptotyphlopidae	1	3	0,2,0,0,0,0	9.5	3,0,0,0
		Natricidae	3	11	0,8,0,0,1,0	11.8	4,1,6.0
		Viperidae	2	11	0,9,0,1,0,0	12.1	1,9,0,0
	**Subtotal**		**65**	**137**	**7,91,2,5,1,0**	**11.99**	**78,32,23,1**
**TOTAL**			**81**	**175**	**10,119,6,6,1,0**	**11.45**	**105,41,25,1**

### General distribution

**Figure 2.**
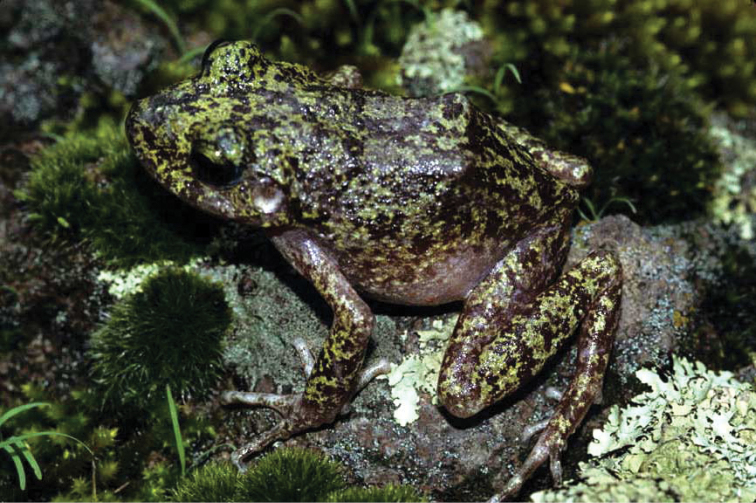
*Craugastor tarahumaraensis*. Ocampo, Chihuahua. Photo courtesy of Peter Heimes.

Thirteen of the 38 species of amphibians that inhabit Chihuahua are endemic to Mexico, one of them (*Lithobates lemosespinali*) is restricted to a small area in the Sierra Madre Occidental of Chihuahua, and another (*Isthmura sierraoccidentalis*) is found only in an isolated population in the Sierra Madre Occidental of Sonora and Chihuahua. Eleven more are distributed in western Mexico (*Ambystoma rosaceum*, *Ambystoma silvense*, *Anaxyrus mexicanus*, *Incilius mazatlanensis*, *Incilius mccoyi*, *Craugastor tarahumaraensis* [Fig. 2], *Eleutherodactylus interorbitalis*, *Agalychnis dacnicolor*, *Tlalocohyla smithii*, *Lithobates magnaocularis*, and *Lithobates pustulosus*). Three more species are widely distributed from southern Canada to northern Mexico (*Ambystoma mavortium*, *Anaxyrus cognatus*, and *Spea bombifrons*). One species (*Lithobates forreri*) is widely distributed from northern Mexico to Central America, with a range that extends from central western Sonora through the Pacific Coast to Costa Rica. Another sixteen species occur from central or southern United States to northern, central or southern Mexico (*Anaxyrus debilis*, *Anaxyrus punctatus*, *Anaxyrus speciosus*, *Anaxyrus woodhousii*, *Incilius alvarius*, *Craugastor augusti*, *Eleutherodactylus marnockii*, *Hyla arenicolor*, *Hyla wrightorum*, *Gastrophryne mazatlanensis*, *Gastrophryne olivacea*, *Lithobates chiricahuensis*, *Lithobates tarahumarae*, *Lithobates yavapaiensis*, *Scaphiopus couchii*, and *Spea multiplicata*). Four more occur from eastern and/or southeastern United States to South America (*Rhinella horribilis*, *Smilisca baudinii*, *Hypopachus variolosus*, and *Lithobates berlandieri*), this last species with isolated populations in the Sierra Madre Occidental. Only one of the amphibian species that currently inhabit Chihuahua was introduced to the state (*Lithobates catesbeianus*).

**Figure 3.**
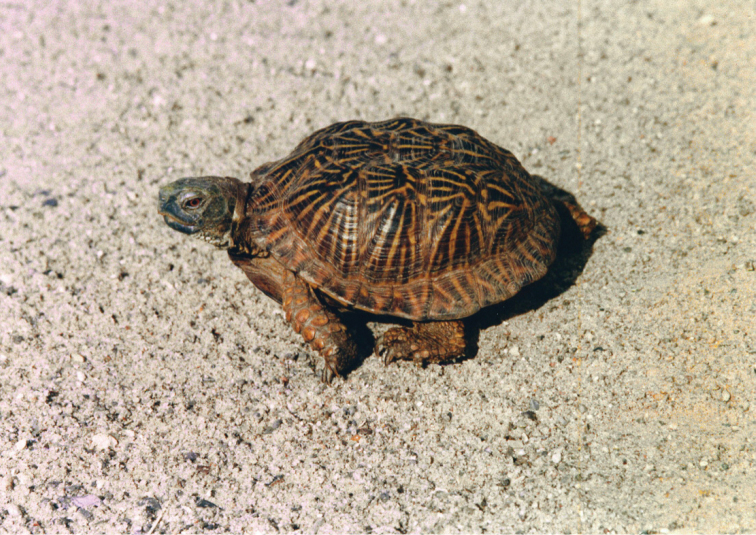
*Terrapene ornata*. Rancho de Flores Magón, Buenaventura, Chihuahua. Photo by Julio Lemos Espinal.

Five of the 13 species of turtles that inhabit Chihuahua are endemic to Mexico, two of them to the Bolsón de Mapimí, a small area in southeastern Chihuahua, southwestern Coahuila, and northeastern Durango (*Kinosternon durangoense* and *Gopherus flavomarginatus*), two more to western Mexico (*Terrapene nelsoni* and *Kinosternon integrum*), and one more to the subtropics of southeastern Sonora, southwestern Chihuahua, and northern Sinaloa (*Gopherus evgoodei*). Six more species occur from central or southern United States to northern (*Terrapene ornata* [Fig. 3], *Trachemys gaigeae*, *Kinosternon flavescens*, and *Kinosternon sonoriense*) or central or southern Mexico (*Kinosternon hirtipes* and *Apalone spinifera*). The remaining two species of turtles are widely distributed from southern Canada to northern Mexico (*Chrysemys picta*) and from southeastern Sonora, through the Pacific Coast, to Costa Rica (*Rhinoclemmys pulcherrima*).

**Figure 4.**
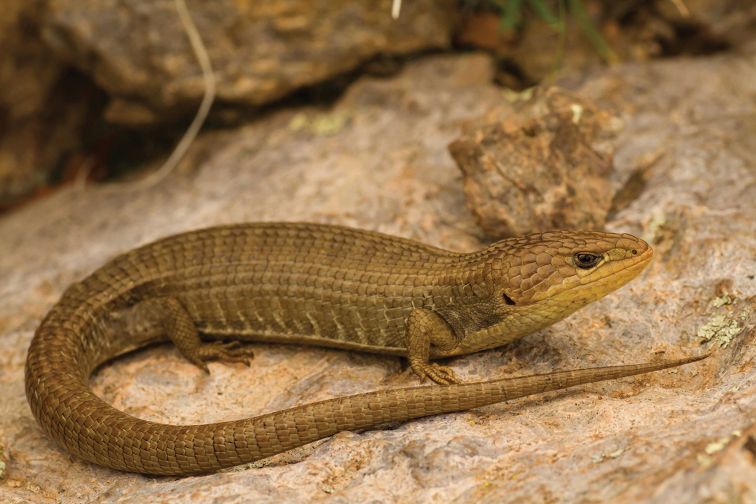
*Barisia levicollis* (female). Sierra del Nido, Chihuahua. Photo courtesy of Marisa Ishimatsu.

Fifteen of the 51 species of lizards that occur in Chihuahua are endemic to Mexico, two of them to the state of Chihuahua (*Barisia levicollis* [Fig. 4] and *Plestiodon multilineatus*), one of the remaining 13 endemics is limited to the Bolsón de Mapimí (*Uma paraphygas*), one more to a small area in eastern Sonora and western Chihuahua (*Sceloporus lemosespinali*), another one to the temperate forests of western Chihuahua and northern Durango (*Plestiodon bilineatus*), one more to the Chihuahua Desert from northern Chihuahua to central Mexico (*Holbrookia approximans*), two others occupy areas in the Sierra Madre Occidental and the Sierra Madre Oriental (*Barisia ciliaris*), and even the Transvolcanic Belt (*Phrynosoma orbiculare*). The remaining seven endemic species are distributed mainly along the Pacific Coast of Mexico (*Anolis nebulosus*, *Ctenosaura macrolopha*, *Sceloporus albiventris*, *Sceloporus nelsoni*, *Urosaurus bicarinatus*, *Plestiodon parviauriculatus*, and *Aspidoscelis costata*).

The remaining 36 lizard species are not endemic to Mexico, one of them is distributed from southern Canada to northern Mexico (*Phrynosoma hernandesi*), and two more range from Mexico to northern Guatemala (*Heloderma horridum*) or to Costa Rica (*Phyllodactylus tuberculosus*) mainly on the Pacific Coast. Another 32 are distributed in the United States and Mexico, most of them are species characteristic of the Chihuahua Desert or woodlands of the Sierra Madre Occidental (*Elgaria kingii*, *Gerrhonotus infernalis*, *Crotaphytus collaris*, *Gambelia wislizenii*, *Coleonyx brevis*, *Cophosaurus texanus*, *Holbrookia elegans*, *Holbrookia maculata*, *Phrynosoma cornutum*, *Phrynosoma modestum*, *Sceloporus bimaculosus*, *Sceloporus clarkii*, *Sceloporus consobrinus*, *Sceloporus cowlesi*, *Sceloporus jarrovii*, *Sceloporus merriami*, *Sceloporus poinsettii*, *Sceloporus slevini*, *Sceloporus virgatus*, *Urosaurus ornatus*, *Uta stansburiana*, *Plestiodon callicephalus*, *Plestiodon multivirgatus*, *Plestiodon obsoletus*, *Plestiodon tetragrammus*, *Aspidoscelis exsanguis*, *Aspidoscelis gularis*, *Aspidoscelis inornata*, *Aspidoscelis marmorata*, *Aspidoscelis sonorae*, *Aspidoscelis tesselata*, and *Aspidoscelis uniparens*). Only one of the 51 lizard species that occur in Chihuahua is an introduced species (*Hemidactylus turcicus*).

**Figure 5.**
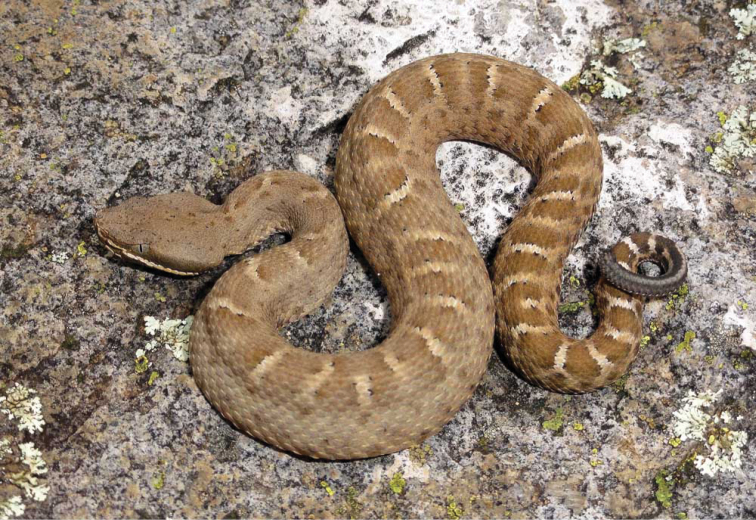
*Crotalus willardi*. Sierra del Nido, Chihuahua. Photo courtesy of Robert Bryson.

Twenty-one of the 73 species of snakes are endemic to Mexico (*Conopsis nasus*, *Leptophis diplotropis*, *Mastigodryas cliftoni*, *Pituophis deppei*, *Salvadora bairdii*, *Sonora aemula*, *Sympholis lippiens*, *Trimorphodon tau*, *Geophis dugesii*, *Lampropeltis polyzona*, *Leptodeira splendida*, *Rhadinaea hesperia*, *Rhadinaea laureata*, *Tropidodipsas repleta*, *Micrurus distans*, *Storeria storerioides*, *Thamnophis errans*, *Thamnophis melanogaster*, *Thamnophis unilabialis*, *Thamnophis validus*, and *Crotalus basiliscus*). Thirty-seven snake species that are found in Chihuahua are distributed from the United States to Mexico (*Arizona elegans*, *Bogertophis subocularis*, *Gyalopion canum*, *Gyalopion quadrangulare*, *Lampropeltis getula*, *Lampropeltis knoblochi*, *Masticophis bilineatus*, *Masticophis flagellum*, *Masticophis taeniatus*, *Pantherophis emoryi*, *Rhinocheilus lecontei*, *Salvadora deserticola*, *Salvadora grahamiae*, *Sonora semiannulata*, *Tantilla hobartsmithi*, *Tantilla nigriceps*, *Tantilla wilcoxi*, *Tantilla yaquia*, *Trimorphodon vilkinsonii*, *Heterodon kennerlyi*, *Hypsiglena chlorophaea*, *Hypsiglena jani*, *Micruroides euryxanthus*, *Rena dissecta*, *Rena humilis*, *Rena segrega*, *Nerodia erythrogaster*, *Thamnophis eques*, *Agkistrodon contortrix*, *Crotalus atrox*, *Crotalus lepidus*, *Crotalus molossus*, *Crotalus ornatus*, *Crotalus pricei*, *Crotalus scutulatus*, *Crotalus viridis*, and *Crotalus willardi* [Fig. 5]). Another four species range from northern Mexico to Central or even South America (*Boa sigma*, *Masticophis mentovarius*, *Imantodes gemmistratus*, and *Agkistrodon bilineatus*). Six more species are found from central or southern United States to Central or South America (*Drymarchon melanurus*, *Drymobius margaritiferus*, *Oxybelis aeneus*, *Senticolis triaspis*, *Thamnophis cyrtopsis*, and *Thamnophis marcianus*). Five more range from Canada to northern or central Mexico (*Opheodrys vernalis*, *Pituophis catenifer*, *Diadophis punctatus*, *Thamnophis elegans*, and *Thamnophis sirtalis*).

In terms of habitat types, 47 species are found in the temperate forests of the Sierra Madre Occidental. Forty-four are found in the subtropical canyons of the Sierra Madre Occidental. Fifty-eight species are found in the Chihuahuan Desert. One species is found in SON. Twenty-five species occupy more than one habitat type (i.e., are generalists).

### Likely species and poorly documented species

There are several additional species that are likely to occur in Chihuahua, but that have not been recorded within the state. Three species of anurans might occur in the deep canyons and lowlands of the extreme southwestern part of the state. The Pacific Stream Frog (*Craugastor vocalis*) was recorded by Hardy and McDiarmid (1969) in extreme northeastern Sinaloa, 16 km NNE Choix, 520 m, near the state line with Chihuahua. The Sabinal Frog (*Leptodactylus melanonotus*) was recorded by Bogert and Oliver (1945) from Güirocoba and Álamos, Sonora, only about 25 and 35km respectively from the Chihuahua border, and by Smith et al. (2005) from the Río Mayo at the gates of Presa Mocuzari, Sonora. Hardy and McDiarmid (1969) mapped localities for this species (as *Leptodactylus occidentalis*, a junior synonym) from throughout the lowlands of Sinaloa, including a locality in the extreme northeastern corner. The Lowland Burrowing Treefrog (*Smilisca fodiens*) has been recorded close to Chihuahua by Hardy and McDiarmid (1969) for Sinaloa, Bogert and Oliver (1945) for Sonora, and Trueb (1969) and Duellman (2001) for both states. Another anuran species likely to occur in extreme northeastern Chihuahua is the Gulf Coast Toad (*Incilius nebulifer*). This species of toad is represented by isolated populations at the southern extremity of the Big Bend region of Texas, adjacent to Coahuila (Conant and Collins 1998).

It is likely that at least four other turtle species occur in Chihuahua. Three species have been taken close to the state line with Sonora and Sinaloa, in the extreme southwestern part of the state. *Kinosternon alamosae* has been taken in the vicinity of Álamos, Sonora, about 35 km from the Chihuahua border. *Trachemys hiltoni* has been recorded from Güirocoba, ~25 km from Chihuahua, and from extreme northern Sinaloa (Hardy and McDiarmid 1969). Seidel (2002) mapped its range into Chihuahua, but only conjecturally. Legler and Webb (1970) stated that the species is limited to the Río El Fuerte drainage. These last authors stated that *Trachemys yaquia* is limited to the drainages of the Río Mayo, Río Sonora and Río Yaqui, however, Seidel (2002) conjectured that the range of this species extended into Chihuahua. In addition, the Common Snapping Turtle (*Chelydra serpentina*) occurs in the Río Grande at least in New Mexico (Degenhardt et al. 1996), and may well occur farther south in extreme northeastern Chihuahua, where little turtle trapping has been done.

There are at least nine lizard species not yet recorded in the state of Chihuahua that are likely to occur in it; four of them in the deep canyons and lowlands of extreme southwestern Chihuahua; three in the extreme northeastern part of the state; and two in the extreme northwestern part. The Zebra-tailed Lizard (*Callisaurus draconoides*) was recorded by Bogert and Oliver (1945) from Güirocoba and Álamos, Sonora (~25 and 35 km respectively from the Chihuahua border), and Hardy and McDiarmid (1969) spotted it at several localities in extreme northeastern Sinaloa. The Black Banded Gecko (*Coleonyx fasciatus*) has been recorded from five localities along the foothills of the Sierra Madre Occidental of eastern Sonora, three of these localities are in the Álamos region, one fairly close to Chihuahua. Its habitat suggests that it might occur in some of the deep canyons of southwestern Chihuahua. The Regal Horned Lizard (*Phrynosoma solare*) ranges from southern Arizona through almost all of Sonora, into northern Sinaloa. Hardy and McDiamid (1969) and Bogert and Oliver (1945) recorded it near Chihuahua in both Sinaloa and Sonora. It is a species of arid and semiarid habitats on plains, hills, and low mountain slopes. The Desert Spiny Lizard (*Sceloporus magister*) shows a range similar to that of the preceding species. East of the Sea of Cortés, it is the western representative of the eastern *Sceloporus bimaculosus*.

In northeastern Chihuahua the presence of three additional lizard species is likely. Wright (1971) indicated that the New Mexico Whiptail (*Aspidoscelis neomexicana*) is known from only central New Mexico and extreme southwestern Texas; almost all records are from near the Río Grande. He projected its range into Chihuahua along the Río Grande; although there are no records, its occurrence is highly likely there. Conant and Collins (1998) depicted the southern part of the Big Bend region of Texas as part of the range of the Reticulate Banded Gecko (*Coleonyx reticulatus*). It may be expected in adjacent parts of Chihuahua. Also Conant and Collins (1998) projected the range of the Texas Alligator Lizard (*Gerrhonotus infernalis*) to include the southern part of the Big Bend region of Texas, southward through eastern Chihuahua, most of Coahuila and other states to the south. In northwestern Chihuahua the presence of the Western Banded Gecko (*Coleonyx variegatus*) is expected. As indicated in Stebbins (2003) and Degenhardt et al. (1996), this species occurs in extreme southwestern New Mexico, and probably also in adjacent northwestern Chihuahua. The Gila Monster (*Heloderma suspectum*) is also expected to occur in this part of the state. The known occurrence of this species in Sonora, Arizona, and New Mexico close to the Chihuahua border indicates that occurrence in Chihuahua is likely.

It is highly likely that nine more snake species occur within the state of Chihuahua. Two of them in southwestern Chihuahua (*Phyllorhynchus browni* and *Pseudoficimia frontalis*); four in northeastern Chihuahua (*Coluber constrictor*, *Lampropeltis alterna*, *Pantherophis bairdi*, *Tantilla cucullata*); two in the northwestern part of the state (*Crotalus tigris*, *Sistrurus catenatus*); and one in extreme southeastern Chihuahua (*Tantilla atriceps*). The Saddled Leaf-nosed Snake (*Phyllorhynchus browni*) was recorded by Bogert and Oliver (1945) from Alamos, ~35 km from the Chihuahua border; Hardy (1972) reviewed the distribution of The False Ficimia (*Pseudoficimia frontalis*), citing specimens from near Álamos and Güirocoba, Sonora, ~35 and 25 km from the Chihuahua border, respectively. The North American Racer (*Coluber constrictor*) is rare in Mexico, with only three records. Two are from Coahuila, including one from the extreme northwestern corner, in the Sierra del Carmen (Wilson 1966). Occurrence in Chihuahua seems likely. The Gray-banded Kingsnake (*Lampropeltis alterna*) is well known in the Big Bend of Texas, and elsewhere in that state, as well as in Coahuila and other adjacent states in Mexico, but it has never been found in Chihuahua, although it almost certainly occurs there. Baird´s Ratsnake (*Pantherophis bairdi*) occurs in western Texas, including the Big Bend region, as well as northern Coahuila (Conant and Collins 1998); it is highly likely to occur in adjacent Chihuahua. The Trans-Pecos Blackheaded Snake (*Tantilla cucullata*) is known only in Texas, in the Big Bend and immediate vicinity (Dixon et al. 2000); occurrence in adjacent Coahuila and Chihuahua is to be expected. In northwestern Chihuahua the occurrence of the Tiger Rattlesnake (*Crotalus tigris*) is expected. Stebbins (2003) indicates occurrence of this species in the extreme southeastern corner of Arizona, and in eastern Sonora near the Chihuahua border. An inhabitant of arid and semiarid foothills deserts, it may enter the latter state in some of its semiarid valleys. Another rattlesnake, the Massasagua (*Sistrurus catenatus*), is known from southern New Mexico (Degenhardt et al. 1996) and southeastern Arizona (Brennan and Holycross 2006); it likely occurs in adjacent Chihuahua. In extreme southeastern Chihuahua the occurrence of the Mexican Black-headed Snake (*Tantilla atriceps*) is expected. The known range of this species comes close to the southeastern corner of the state (Cole and Hardy 1981, Conant and Collins 1998).

Some amphibian and reptile species are known to occur in Chihuahua from only a few records, including the Sonoran Desert Toad (*Incilius alvarius*) recorded by Santos-Barrera et al. (2006) in the municipality of Janos; the Spectacled Chirping Frog (*Eleutherodactylus interorbitalis*) recorded by Lemos-Espinal et al. (2006) in Cumbre del Caballo, Chínipas; the Cliff Chirping Frog (*Eleutherodactylus marnockii*) recorded by Lemos-Espinal et al. (2001) in the Grutas de Coyame; the Many-lined Skink (*Plestiodon multivirgatus*) recorded only by Van Devender and Van Devender (1975) at Ojo de Galeana; the Smooth Green Snake (*Opheodrys vernalis*) recorded only by Van Devender and Lowe (1977) at 38.4 km SE of Guerrero; the Crowned Graceful Brown Snake (*Rhadinaea laureata*) recorded by Villa et al. (2012) near km 86 on Hwy 25 N of Creel, Bocoyna, and 1 km N of Baborigame, Guadalupe y Calvo; the Banded Blacksnake (*Tropidodipsas repleta*) recorded by H. Smith and Lemos-Espinal (2006) at km 36 road Temoris-Chínipas, Guazapares; and the Plain-bellied Watersnake (*Nerodia erythrogaster*) recorded by Uriarte-Garzón and García-Vázquez (2014) in the municipality of Ojinaga.

### Comparisons with neighboring states

Overall, the species of amphibians and reptiles in Chihuahua represent just over 37% of the total pool of species from Chihuahua and its neighboring states (Tables 3, 4). Species of reptiles from Chihuahua make up even more of the total pool of species, especially the Squamata, and more specifically Anguids and Snakes. Chihuahuan amphibians make up less of the species pool, especially salamanders. Chihuahua has a good proportion of the region’s Ambystomatid salamanders, but is very depauperate in Plethodontids.

**Table 3.** Total number of native amphibian and reptile species in each state arranged according to taxonomic order/suborder. Superscripts indicate number of introduced species to the state.

Order/Suborder	Chihuahua	New Mexico	Texas	Sonora	Sinaloa	Durango	Coahuila
Caudata	4	3	28	3	1	3	4
Anura	33^1^	23^1^	41^1^	33^2^	35	30^1^	20
Crocodilia			1	1	1		
Testudina	13	10	30^1^	16^1^	12	5	11
Squamata/Lacertilia	50^1^	46^1^	45^6^	66^3^	35	49^1^	49^1^
Squamata/Serpentes	73	52	75^2^	71^1^	62	59^1^	49
**TOTAL**	**173^2^**	**134^2^**	**220^10^**	**190^7^**	146*	**146^3^**	**133^1^**

*Introduced species for the state of Sinaloa are not documented in Enderson et al. (2009).

Overall, Chihuahua shares the highest proportion of its species with Sonora followed by Durango (Table 4). This is particularly evident in amphibians, with over 80% of Chihuahuan amphibians shared with Sonora. For reptiles, Chihuahua shares nearly 77% of its species with Durango and 66% with Sonora. Chihuahua generally shares the least number of species with Coahuila, Sinaloa, and Texas. These patterns of shared species are likely a function of the extent to which these states share habitat types. For example, Chihuahua, Sonora, and Durango all have extensive desert habitats whereas Texas, for example, has a much more diverse range of habitats than Chihuahua. In addition, Sonora and Chihuahua share the habitats and species found in the Sierra Madre Occidental. Our results considering Chihuahua and all of its neighboring states parallels the results of an analysis of the states along the US-Mexico border using Jaccard hierarchical clustering analyses (Smith and Lemos-Espinal 2015).

**Table 4.** Summary of the numbers of species shared between Chihuahua and neighboring Mexican and American states (not including introduced species). The percent of Chihuahuan species shared by a neighboring state are given in parentheses. Total refers to the total number of species found in Chihuahua and all the neighboring states (i.e., regional species pool) and the number in parentheses in this column is the percent of the regional species pool found in Chihuahua. -- indicates either Chihuahua or the neighboring state has no species in the taxonomic group, thus no value for shared species is provided.

	Chihuahua	New Mexico	Texas	Sonora	Sinaloa	Durango	Coahuila	Total
Class Amphibia	37	17 (45.9)	17 (45.9)	30 (81.1)	20 (54.0)	23 (62.2)	15 (40.5)	122 (30.1)
Order Caudata	4	1 (25)	1 (25)	3 (75)	1 (25)	3 (75)	1 (25)	36 (11.1)
Ambystomatidae	3	1 (33.3)	1 (33.3)	2 (66.7)	1 (33.3)	3 (100)	1 (33.3)	8 (37.5)
Amphiumidae	0	–	–	–	–	–	–	1 (0)
Plethodontidae	1	0 (0)	0 (0)	1 (100)	–	–	–	22 (4.5)
Proteidae	0	–	–	–	–	–	–	1 (0)
Salamandridae	0	–	–	–	–	–	–	2 (0)
Sirenidae	0	–	–	–	–	–	–	2 (0)
Order Anura	33	16 (48.5)	16 (48.5)	27 (81.8)	19 (57.6)	20 (60.6)	14 (42.4)	86 (38.4)
Bufonidae	10	6 (60)	6 (60)	9 (90)	6 (60)	8 (80)	6 (60)	21 (47.6)
Craugastoridae	2	1 (50)	1 (50)	2 (100)	1 (50)	2 (100)	1 (50)	5 (40)
Eleutherodactylidae	2	–	1 (50)	1 (50)	1 (50)	0 (0)	1 (50)	10 (20)
Hylidae	5	2 (40)	2 (40)	5 (100)	4 (80)	3 (60)	2 (40)	22 (22.7)
Leptodactylidae	0	–	–	–	–	–	–	2 (0)
Microhylidae	3	1 (33.3)	2 (66.7)	2 (66.7)	2 (66.7)	1 (33.3)	1 (33.3)	5 (60)
Ranidae	8	3 (37.5)	1 (12.5)	6 (75)	4 (50)	4 (50)	1 (12.5)	16 (50)
Rhinophrynidae	0	–	–	–	–	–	–	1 (0)
Scaphiopodidae	3	3 (100)	3 (100)	2 (66.7)	1 (33.3)	2 (66.7)	2 (66.7)	4 (75)
Class Reptilia	136	76 (55.9)	66 (48.5)	90 (66.2)	61 (44.8)	86 (76.8)	62 (59.6)	343 (39.6)
Order Crocodylia	0	–	–	–	–	–	–	2 (0)
Crocodylidae	0	–	–	–	–	–	–	2 (0)
Order Testudines	13	6 (46.2)	6 (46.2)	6 (46.2)	4 (30.8)	5 (38.5)	6 (46.2)	47 (27.6)
Chelonidae	0	–	–	–	–	–	–	5 (0)
Chelydridae	0	–	–	–	–	–	–	2 (0)
Dermochelyidae	0	–	–	–	–	–	–	1 (0)
Emydidae	4	3 (75)	3 (75)	2 (50)	1 (25)	1 (25)	1 (25)	22 (18.2)
Geomydidae	1	–	–	1 (100)	1 (100)	–	–	1 (100)
Kinosternidae	5	2 (40)	2 (40)	2 (40)	1 (20)	3 (60)	3 (60)	10 (50)
Testudinidae	2	–	0 (0)	1 (50)	1 (50)	1 (50)	1 (50)	4 (5)
Trionychidae	1	1 (100)	1 (100)	–	–	–	1 (100)	2 (50)
Order Squamata	123	70 (56.9)	60 (48.8)	84 (68.3)	57 (46.3)	81 (65.8)	56 (45.5)	294 (41.8)
Suborder Lacertilia	50	30 (60)	25 (50)	33 (66)	17 (34)	34 (68)	23 (46)	143 (35)
Anguidae	4	1 (25)	1 (25)	1 (25)	1 (25)	3 (75)	2 (50)	7 (57.1)
Crotaphytidae	2	2 (100)	2 (100)	2 (100)	–	2 (100)	2 (100)	6 (33.3)
Dactyloidae	1	–	0 (0)	1 (100)	1 (100)	1 (100)	–	4 (25)
Eublepharidae	1	1 (100)	1 (100)	0 (0)	0 (0)	1 (100)	1 (100)	4 (25)
Helodermatidae	1	0 (0)	–	1 (100)	1 (100)	1 (100)	–	2 (50)
Iguanidae	1	–	–	1 (100)	1 (100)	0 (0)	–	9 (11.1)
Phrynosomatidae	24	16 (66.7)	12 (50)	19 (79.2)	9 (37.5)	18 (75)	12 (50)	61 (39.3)
Phyllodactylidae	1	–	–	1 (100)	1 (100)	1 (100)	–	5 (20)
Scincidae	7	3 (42.8)	3 (42.8)	3 (42.8)	2 (28.6)	3 (42.8)	2 (28.6)	18 (39.9)
Teiidae	8	7 (87.5)	6 (75)	4 (50)	1 (12.5)	4 (50)	4 (50)	23 (34.8)
Xantusidae	0	–	–	–	–	–	–	4 (0)
Suborder Serpentes	73	40 (54.8)	35 (47.9)	51 (69.9)	40 (54.8)	47 (64.4)	33 (45.2)	151 (48.3)
Boidae	1	–	–	1 (100)	1 (100)	1 (100)	–	2 (50)
Colubridae	35	21 (60)	19 (54.3)	24 (68.6)	22 (62.8)	25 (71.4)	17 (48.6)	66 (53)
Dipsidae	10	4 (40)	3 (30)	7 (70)	6 (60)	6 (60)	3 (30)	22 (45.4)
Elapidae	2	1 (50)	0 (0)	2 (100)	2 (100)	–	0 (0)	4 (50)
Leptotyphlopidae	3	2 (66.7)	2 (66.7)	1 (33.3)	1 (33.3)	1 (33.3)	2 (66.7)	4 (75)
Natricidae	11	6 (54.5)	4 (36.4)	7 (63.6)	3 (27.3)	9 (81.8)	3 (27.3)	32 (34.4)
Viperidae	11	6 (54.5)	7 (63.6)	9 (81.8)	5 (45.4)	5 (745.4)	8 (72.7)	21 (52.4)
**TOTAL**	**173**	**93 (53.8)**	**83 (48.0)**	**120 (69.4)**	**81 (46.8)**	**109 (63.0)**	**77 (44.5)**	**465 (37.2)**

### Conservation status

Most of the herpetofauna of Chihuahua falls in the IUCNs least concern category (119 of 132 [does not include DD species]; 90%), and as not listed by SEMARNAT (105 of 172; 61%) (Table 2). These percentages are similar to those from other recently compiled tallies of conservation statuses for Mexican states (Coahuila: Lemos-Espinal and G. Smith 2016, Hidalgo: Lemos-Espinal and G. Smith 2015, Nayarit: Woolrich-Piña et al. 2016, Nuevo León: Lemos-Espinal et al. 2016). However, there are species of conservation concern in Chihuahua. For example, turtles and tortoises in Chihuahua appear to be a group of particular conservation concern with nearly half considered Vulnerable or Near Threatened by IUCN and more than half listed as Pr or A by SEMARNAT. Emydidae and Testudinae are the families of most conservation concern. Indeed, turtles account for 4 of the 13 species (31%) of the Chihuahuan herpetofauna that are categorized as Vulnerable, Near Threatened, or Endangered by the IUCN, even though they make up only 7% of the species in Chihuahua. We also found that turtles as a group also have the highest mean Environmental Vulnerability Score (EVS), especially Emydidae, Testudinidae, and Trionychidae. We therefore encourage additional emphasis be placed on better understanding the ecology and conservation status of turtle and tortoise populations in Chihuahua.

In addition, even though there are relatively few reptiles and amphibians placed on conservation lists in Chihuahua, this does not mean they are safe. Indeed, there are species, such as *Craugastor tarahumaraensis*, *Ctenosaura macrolopha*, *Uma paraphygas*, and *Tropidodipsas repleta* that are of great conservation concern based on their EVS values (Wilson et al. 2013a, b). In addition, the more locally appropriate EVS assessments (see Wilson et al. 2013a,b) also suggest that conservation concern should exist for the amphibian families Ambystomatidae, Craugastoridae, and Eleutherodactylidae; and the non-turtle reptile families Anguidae, Eublepharidae, Iguanidae, Scincidae, Teiidae, and Elapidae.

Even beyond these species and families, the environment and habitats of Chihuahua are subject to anthropogenic change, such as construction of border fences (Lasky et al. 2011), increased urbanization (Biggs et al. 2010), and changes in precipitation and increased drying associated with climate change (Seager and Vecchi 2010). Indeed, the distribution of species at high risk according to the EVS assessment (≥ 14; Wilson et al. 2013a,b) is not the same across habitat types. Nearly 40% of species (18/47) in the temperate forests of the Sierra Madre Occidental is at risk according to the EVS, and nearly a third of species in the Chihuahuan Desert (19/58). Just over 20% (10/44) of species in subtropical canyons of the Sierra Madre Occidental are at high risk. Generalist species (those that use more than one habitat type) are the least at risk (2 of 25 species). These results suggest that particular conservation attention should be paid to the Sierra Madre Occidental and the Chihuahuan Desert habitat types in Chihuahua. We thus again emphasize that continued and increased study of the herpetofauna of Chihuahua is needed to monitor the possible effects of any environmental changes.
